# Integrated omics analysis on organic fertilizer-mediated regulation of starch synthesis in cassava

**DOI:** 10.3389/fpls.2026.1821836

**Published:** 2026-06-01

**Authors:** Jiongyu Chen, Zizhou Huang, Lijun Wei, Kaiwen Lei, Di Wang, Ruirui Chen, Ping Lv, Benchi Yu, Saiqing Lu

**Affiliations:** 1 Guangxi Subtropical Crops Research Institute, Nanning, Guangxi, China; 2 Guangxi Key Laboratory of Quality and Safety Control for Subtropical Fruits, Guangxi Subtropical Crops Research Institute, Nanning, Guangxi, China

**Keywords:** cassava, metabolome, organic fertilizer, starch synthesis, transcriptome

## Abstract

Cassava (*Manihot esculenta* Crantz) is a vital food security crop and industrial feedstock worldwide. Although the excessive use of chemical fertilizers threatens soil health and sustainable agriculture, organic amendments provide an environmentally friendly alternative. In this study, organic fertilizer (chicken manure) was set as treatment and chemical fertilizer as control. The regulatory mechanisms of mature cassava starch synthesis were initially elucidated by integrating agronomic traits, physiological indices, and transcriptome-metabolome analyses. The results showed that organic fertilizer significantly enlarged the leaf xylem cell area, effectively promoting nutrient uptake and utilization. It also markedly improving leaf photosynthetic assimilation and root starch synthesis. Ultimately, organic fertilizer increased cassava yield by 13.72%-26.08% and starch content by 9.90%-11.21%. Combined omics analysis revealed that organic fertilizer modulates stress-related pathways through multiple mechanisms, including elevated levels of caffeoylshikimic acid and caffeoylquinic acid, upregulating genes related to glutathione metabolism, and downregulating β-1, 3-glucanase genes. In conclusion, organic fertilizer promotes photosynthetic assimilation and starch synthesis in cassava through multiple mechanisms. These effects lead to simultaneous increases in storage root yield and starch content.

## 1 Introduction

Cassava (*Manihot esculenta* Crantz) is a vital food and industrial raw material crop in tropical and subtropical regions, and its yield and quality directly affect industrial profitability. As the primary indicator of cassava quality and processing value, starch content is strategically important for advancing key fields such as regional food security, bioenergy production, and food processing (Chisenga et al., 2019; Zhou et al., 2020; Lu et al., 2025). However, frequent extreme weather events driven by global climate change subject cassava production to combined stresses, including drought, waterlogging, pests, and diseases. These stresses reduce tuber and starch yields and severely hinder industrial development (Devi et al., 2022). Therefore, exploring stable and effective agronomic practices and biological mechanisms to increase starch content has become one of the most urgent core issues in cassava production.

Over the long term, chemical fertilizers have played a key role in high crop yields (Jiang et al., 2019). However, long-term reliance on and excessive use of chemical fertilizers tend to cause soil compaction, acidification, microbial community imbalances, and non-point source pollution, thereby damaging the soil ecosystem and posing potential threats to crop yield and quality (Agbede, 2010). In recent years, the substitution of chemical fertilizers with organic fertilizers, either partially or entirely, has become a key strategy in the green transformation of agriculture (Shi et al., 2023). Numerous studies have demonstrated that organic fertilizers have positive effects on both crop yield and quality. For example, organic fertilizers can significantly enhance the quality indicators of tea (Chen et al., 2022). Additionally, it can increase the yield of black rice by 5.15 t/ha, raise its starch content by 33.86%, and boost its anthocyanin content by 275.18 ppm (Herliana et al., 2018).

Starch synthesis is a fundamental metabolic process essential for plant growth and development (MacNeill et al., 2017). This process begins with the synthesis of sucrose in the leaves via photosynthesis (De Souza et al., 2020). Sucrose is then transported over long distances to storage organs through the phloem, where starch biosynthesis is ultimately completed (Sauer, 2007; Ruan et al., 2010). Through sucrose transport and a series of enzymatic reactions, photosynthetic products are gradually converted into amylose and amylopectin (Comparot-Moss and Denyer, 2009; Tappiban et al., 2019; He et al., 2024). The efficiency of these processes directly determines the level of starch accumulation in plants. Numerous studies have demonstrated that organic fertilizers can increase starch content in plants by enhancing photosynthetic capacity and improving carbon metabolism (Wang et al., 2022; Wei et al., 2024b; Han et al., 2025). Among various organic fertilizers, chicken manure has received extensive attention due to its comprehensive nutrient composition, high organic matter content, and its effectiveness in improving the soil microecological environment. Additionally, its role in enhancing starch content in plants has been reported across a variety of crops. For example, studies have shown that the sole application of chicken manure significantly increased the starch content of potatoes, with the highest starch content observed when chicken manure was combined with mycorrhizal fungus (Abdulkadhum et al., 2023). Moreover, research has found that the application of lime mixed with chicken manure ash significantly enhanced the starch content in ponkan leaves (Zhang et al., 2025). In cassava cultivation, the application of organic fertilizer to enhance starch content has been initially explored. For example, research has demonstrated that chicken manure could enhance fresh tuber yield and starch yield of cassava in low productivity sandy soil (Jindaluang et al., 2025). Meanwhile, our previous study compared the effects of different organic fertilizers on cassava yield and starch content, and the results showed that chicken manure had the most significant positive impact on cassava starch accumulation and yield improvement (Wei et al., 2024c). Although the positive effects of organic fertilizers, particularly chicken manure, on improving crop yield and starch content have been well documented, most studies have been limited to phenotypic observations. The underlying physiological and molecular regulatory mechanisms remain poorly understood, and comprehensive multi-omics analyses have yet to be conducted.

In this study, two cassava varieties were used as experimental materials, and organic fertilizer treatments were applied. Combined enzyme activity assays, leaf anatomical structure observations, and integrated transcriptome-metabolome analyses were conducted to systematically elucidate the regulatory effects and underlying mechanisms of organic fertilizer on cassava starch synthesis. The study aims to reveal the mechanisms by which organic fertilizer promotes cassava starch synthesis at both physiological and molecular levels, providing a theoretical foundation and technical support for optimizing organic high-yield cultivation techniques and the genetic improvement of high-starch cassava varieties.

## 2 Materials and methods

### 2.1 Experimental design

This experiment was conducted from April 2024 to December 2024 at the experimental base of the Institute of Subtropical Crops in Nanning, Guangxi Zhuang Autonomous Region, China (22°89′N, 108°34′E). Two cassava varieties, Guire 11 (GR11) and Guire 13 (GR13), were tested. Both varieties were provided by the Guangxi Cassava Research Institute and were planted in April 2024. A split plot design was adopted for the field experiment, with cassava varieties as the main factor and fertilization regimes as the secondary factor. Basal fertilizer was the only experimental variable between treatments, while all other field management practices followed conventional agronomic methods for cassava. Two basal fertilizer treatments were established: the control group (CK) received 50 g of compound fertilizer (N:P:K = 15:15:15) per plant, and the treatment group (T) was applied 50 g of fermented chicken manure per plant. The chicken manure contained the following nutrient contents: moisture 53.75%, total nitrogen (TN) 1.61%, total phosphorus (TP) 1.52%, total potassium (TK) 1.95%, and organic matter 41.17%. It was fully fermented and adjusted to a moisture content of 25%-30% before application. Cassava was planted at a spacing of 1 m × 0.8 m (plant spacing × row spacing).

### 2.2 Determination of agronomic traits

At cassava maturity, agronomic traits were investigated. Ten cassava plants were randomly selected from each experimental plot. For each individual plant, plant height, stem diameter, and fresh weight of tuberous roots (W_1_) were measured. Plant height was defined as the linear distance from the stem base to the top growing point. Stem diameter was determined by averaging two measurements taken at the same position, 50 cm from the ground, with the stem rotated 90° between measurements. Approximately 500 g of tuberous roots was taken, peeled, rinsed, cut into pieces, and weighed (W_2_), then dried at 60 °C to a constant weight (W_3_).

The calculation formulas for yield and dry matter rate are as follows:


$$yield\left(kg/ha\right)=\frac{10000}{1\times 0.8}\times W_{1}$$

Note: 10, 000 refers to 10, 000 m², equivalent to 1 hectare (ha); 1×0.8 represents the plant-row spacing (m); W_1_ is the fresh tuberous root weight per plant (kg).


$$Dry\;matter\;rate\left(\%\right)=\frac{W_{3}}{W_{2}}\times 100\%$$

Note: W_2_ is the fresh weight of the samples before drying (kg); W_3_ is the weight after drying to a constant weight (kg).

The hydrostatic balance technique was employed to determine the starch content in cassava (Peprah et al., 2020; Howeler R, 2014). First, the cassava tuberous roots were thoroughly cleaned to remove any residual soil from the surface. Then, a 5 kg sample was accurately weighed. A container was filled with water, and the root sample was suspended in the water using a support, ensuring that the roots did not touch the inner walls or bottom of the container. The weight of the fully submerged roots was recorded (W_4_), and the starch content was calculated accordingly.

The calculation formula for starch content is as follows:


$$Starch\;content\left(\%\right)=210.8\times \frac{5}{5-W_{4}}-213.4$$

Note: W_4_ is the weight of 5 kg of tuberous roots in water (kg).

### 2.3 Determination of enzyme activity

The fifth fully expanded leaf from the top of cassava at maturity under different treatments was sampled to determine enzyme activity, with three independent biological replicates included for each treatment group. The activities of sucrose synthase (SUS), sucrose phosphate synthase (SPS), ADP-glucose pyrophosphorylase (AGPase), fructose-bisphosphate aldolase (FBA), ribulose-1, 5-bisphosphate carboxylase/oxygenase (Rubisco), and starch branching enzyme (SBE) were measured using commercial enzyme activity assay kits (Beijing Solarbio Science & Technology Co., Ltd., Beijing, China). All procedures were conducted strictly in accordance with the manufacturer’s instructions.

### 2.4 Observation of leaf cell morphology

The fifth fully expanded leaf from the top of GR11 and GR13 plants under different treatments was collected. The middle part of their central lobes was cut into approximately 0.5 cm wide sections and fixed in 50% FAA fixative. Paraffin embedding and sectioning were performed following the method of Xie et al. (2019). The sections were then observed and photographed using a LEICA DM200 LED microscope (Leica Microsystems, Wetzlar, Germany). The length, width, and area of xylem cells were measured by CaseViewer.

### 2.5 Transcriptomic analysis

Compared to GR11, GR13 has a broader promotion area within the local region, is one of the primary cultivated varieties, and demonstrates greater representativeness. Therefore, leaves and tuberous roots of GR13 were selected for subsequent transcriptome and metabolome analyses. At the mature stage, three cassava plants were randomly selected from each group, and leaves (≥1 g) and tuberous roots (≥3 g) were sampled separately. Leaves were rinsed with sterile ultrapure water, blotted dry with absorbent paper, placed into 50 mL centrifuge tubes, and snap-frozen in liquid nitrogen. For the tuberous roots, after peeling off the outer and inner epidermis, the middle flesh was excised, minced, transferred into 50 mL centrifuge tubes, and immediately frozen in liquid nitrogen.

Total RNA was extracted using the E.Z.N.A.^®^ Fungal RNA Mini Kit (Omega Bio-tek, Inc., Norcross, GA, USA). RNA concentration and purity were measured with a NanoDrop 2000 spectrophotometer (Thermo Fisher Scientific, Waltham, MA, USA). RNA integrity was assessed by 1% agarose gel electrophoresis. Transcriptome libraries were constructed using the VAHTS Universal V8 RNA-seq Library Prep Kit for MGI (Vazyme, Nanjing, China) according to the manufacturer’s instructions. Library quality was assessed with an Agilent 2100 Bioanalyzer System (Agilent Technologies, Inc., Santa Clara, CA, USA) and a Qubit Fluorometric Quantitation System (Thermo Fisher Scientific). Qualified libraries were subjected to RNA sequencing using the DNBSEQ-T7 sequencer (MGI Tech Co., Ltd., Shenzhen, China). All RNA purification, reverse transcription, library construction, and sequencing procedures were performed by Wuhan Benagen Technology Co., Ltd. (Wuhan, China).

Raw reads were filtered using fastp (Chen et al., 2018) with default parameters, and the filtered data underwent quality control with FastQC (Davis et al., 2021). Clean reads were aligned to the cassava reference genome index (Manihot_esculenta_v8: GCA_001659605.2. https://plants.ensembl.org/Manihot_esculenta/Info/Index) using STAR (Dobin et al., 2013), and alignment efficiency was calculated and summarized. Transcript assembly was then performed using StringTie (Pertea et al., 2015). Gene expression levels were quantified using RSEM (Li and Dewey, 2011), with FPKM (Fragments Per Kilobase of transcript per Million mapped reads) as the expression metric. The DESeq2 package was used to identify differentially expressed genes (DEGs) (Love et al., 2014). A threshold of *p* ≤ 0.05 and |log_2_ fold change| ≥ 1 was set to determine significant gene expression differences between two samples (Wei et al., 2024a; Zhang et al., 2025). Gene Ontology (GO, https://www.geneontology.org/) and Kyoto Encyclopedia of Genes and Genomes (KEGG, http://www.genome.jp/kegg/) enrichment analyses of the identified DEGs were conducted using topGO and clusterProfiler, respectively.

### 2.6 Metabolomics analysis

The untargeted metabolome was determined by Wuhan Benagen Technology Co., Ltd. The plant samples (20 mg) were lyophilized, mixed with beads, and 1 mL of extraction solution (MeOH: ACN:H2O = 2:2:1, v/v/v) containing deuterated internal standards was added. The mixture was vortexed for 30 s, then homogenized (35 Hz, 4 min) and sonicated for 5 min in a 4 °C water bath, with this step repeated three times. Subsequent to incubation at –40 °C for 1 h, the samples were centrifuged at 12, 000 rpm and 4 °C for 15 min. The supernatant was collected, filtered through a 0.22 μm microporous membrane, and 100 μL of the filtrate was transferred to injection vials for LC-MS/MS analysis. Meanwhile, equal volumes of all samples were mixed to prepare quality control (QC) samples for monitoring system stability and data reliability, with the same processing and detection procedures as the analytical samples.

For non-polar metabolites, LC-MS/MS analyses were performed using an UHPLC system (Vanquish, Thermo Fisher Scientific) equipped with a Phenomenex Kinetex C18 column (2.1 mm × 100 mm, 2.6 μm) coupled to an Orbitrap Exploris 120 mass spectrometer (Orbitrap MS, Thermo Fisher Scientific). The mobile phase consisted of phase A (water with 0.01% acetic acid) and phase B (isopropanol:acetonitrile = 1:1, v/v). Column temperature was 25°C. The autosampler temperature was 4°C, and the injection volume was 2 μL. The Orbitrap Exploris 120 mass spectrometer was used for its ability to acquire MS/MS spectra in information-dependent acquisition (IDA) mode, controlled by the acquisition software (Xcalibur, Thermo). The electrospray ionization (ESI) source conditions were set as follows: sheath gas flow rate at 50 Arb, auxiliary gas flow rate at 15 Arb, capillary temperature at 320 °C, and sweep gas at 1 Arb. Vaporizer temperature: 350°C; full MS resolution: 60, 000; MS/MS resolution: 15, 000; collision energy: stepped normalized collision energy (SNCE) 20/30/40; spray voltage: 3.8 kV (positive) or –3.4 kV (negative), respectively.

After normalizing the original peak areas to the total peak area, subsequent analyses were conducted. Partial least squares discriminant analysis (PLS-DA) was used to maximize the differences in the metabolome between sample pairs. The relative importance of each metabolite in the orthogonal projections to latent structures discriminant analysis (OPLS-DA) model was checked using the variable importance in projection (VIP) parameter. Metabolites with a *p*-value ≤ 0.05 and a VIP score ≥ 1 were considered differentially expressed metabolites (DEMs) between the two samples. Additionally, pathway enrichment analysis was conducted using databases and analytical platforms, including KEGG and MetaboAnalyst (http://www.metaboanalyst.ca/).

### 2.7 Integrated analysis of the transcriptome and metabolome

DEGs and DEMs were jointly mapped to the KEGG pathway database. Pearson correlation coefficients and their corresponding *p*-values were used to identify metabolites and their associated genes within significantly altered KEGG metabolic pathways, and pathway maps were subsequently constructed.

### 2.8 Two-way orthogonal partial least squares model construction and correlation analysis

Based on the transcriptomic and metabolomic data obtained in this study, the O2PLS model was constructed using the OmicsPLS package (version 2.0.2) to perform an integrated analysis of the load values for all significantly differentially expressed genes and metabolites. Load plots for these differentially expressed genes and metabolites were generated (Bylesj et al., 2007). The Pearson correlation coefficient was calculated among agronomic traits, genes, and metabolites, and a Circos chord diagram was plotted to visually illustrate their correlations.

### 2.9 Quantitative real-time polymerase chain reaction validation

To validate the accuracy of the RNA-Seq data, eight genes were randomly selected for qRT-PCRanalysis. The single-strand complementary DNA (cDNA) for qPCR was synthesized using the HiScript^®^ III RT SuperMix for qPCR (+gDNA wiper) Kit (Vazyme). Using *cassava elongation factor 1α (MeEF1α*) as an internal reference gene (Chang et al., 2020; Zheng et al., 2024), specific primers were designed using the NCBI-Primer-BLAST tool (primers are shown in **Supplementary Table 1**). qRT-PCR was performed on a LightCycler 480 PCR instrument (Roche Molecular Systems, Inc., Basel, Switzerland) using ChamQ Universal SYBR qPCR Master Mix (Vazyme), following a two-step amplification program: pre-denaturation at 95 °C for 60 s, denaturation at 95 °C for 10 s, annealing at 60 °C for 30 s, totaling 40 cycles. The relative expression levels of genes were calculated using the 2^-ΔΔCt^ method, with three biological replicates set for each sample.

### 2.10 Statistical analysis

The data were processed using Microsoft Excel 2021 (Microsoft Corporation, Redmond, Washington, USA) and are presented as mean ± standard error (SE). Statistical analysis and data visualization were performed with OriginPro 2026 (OriginLab, Northampton, MA, USA). Multiple comparisons were conducted using Fisher’s least significant difference (LSD) test, and a significant difference between two groups was established at *p* < 0.05.

## 3 Results

### 3.1 Effects of organic fertilizer treatment on agronomic traits of cassava

The results showed that organic fertilizer treatment had a positive regulatory effect on the agronomic traits of both cassava varieties. Specifically, organic fertilizer treatment significantly increased the plant height, starch content, yield, and dry matter rate of GR13 by 1.94%, 9.90%, 26.08%, and 4.23%, respectively, compared to the control group (*p* < 0.05). Its stem diameter was slightly higher than that of the control group, but the difference was not significant. For GR11, organic fertilizer application significantly increased starch content, yield, and dry matter rate by 11.21%, 13.72%, and 4.58%, respectively, compared with the control group (*p* < 0.05). Plant height and stem diameter were slightly higher than those of the control group but did not reach statistical significance (**Table 1**).

**Table 1:** Effects of chicken manure treatment on agronomic traits of cassava.

Variety	Group	Plant height (cm)	Stem diameter (mm)	Starch content (%)	Yield (kg/ha)	Dry matter rate (%)
GR11	CK	132.1 ± 0.61 a	19.34 ± 0.63 a	27.5 ± 0.59 b	47106.88 ± 614.09 b	43.41 ± 0.43 b
	T	134.7 ± 1.24 a	20.65 ± 0.55 a	30.5 ± 0.43 a	53568.44 ± 1985.74 a	45.40 ± 0.47 a
GR13	CK	185.9 ± 0.55 b	24.13 ± 0.25 a	29.3 ± 0.69 b	48274.13 ± 649.84 b	44.64 ± 0.64 b
	T	189.5 ± 0.66 a	25.53 ± 0.69 a	32.2 ± 0.61 a	60863.75 ± 630.85 a	46.53 ± 0.14 a

Values are presented as mean ± standard error (SE). Multiple comparisons were performed using Fisher’s LSD test. Different lowercase letters within the same column indicate significant differences between the CK and T groups (*p* < 0.05).

### 3.2 Effects of organic fertilizer treatment on enzymes activities related to cassava starch synthesis

**Figure 1:**
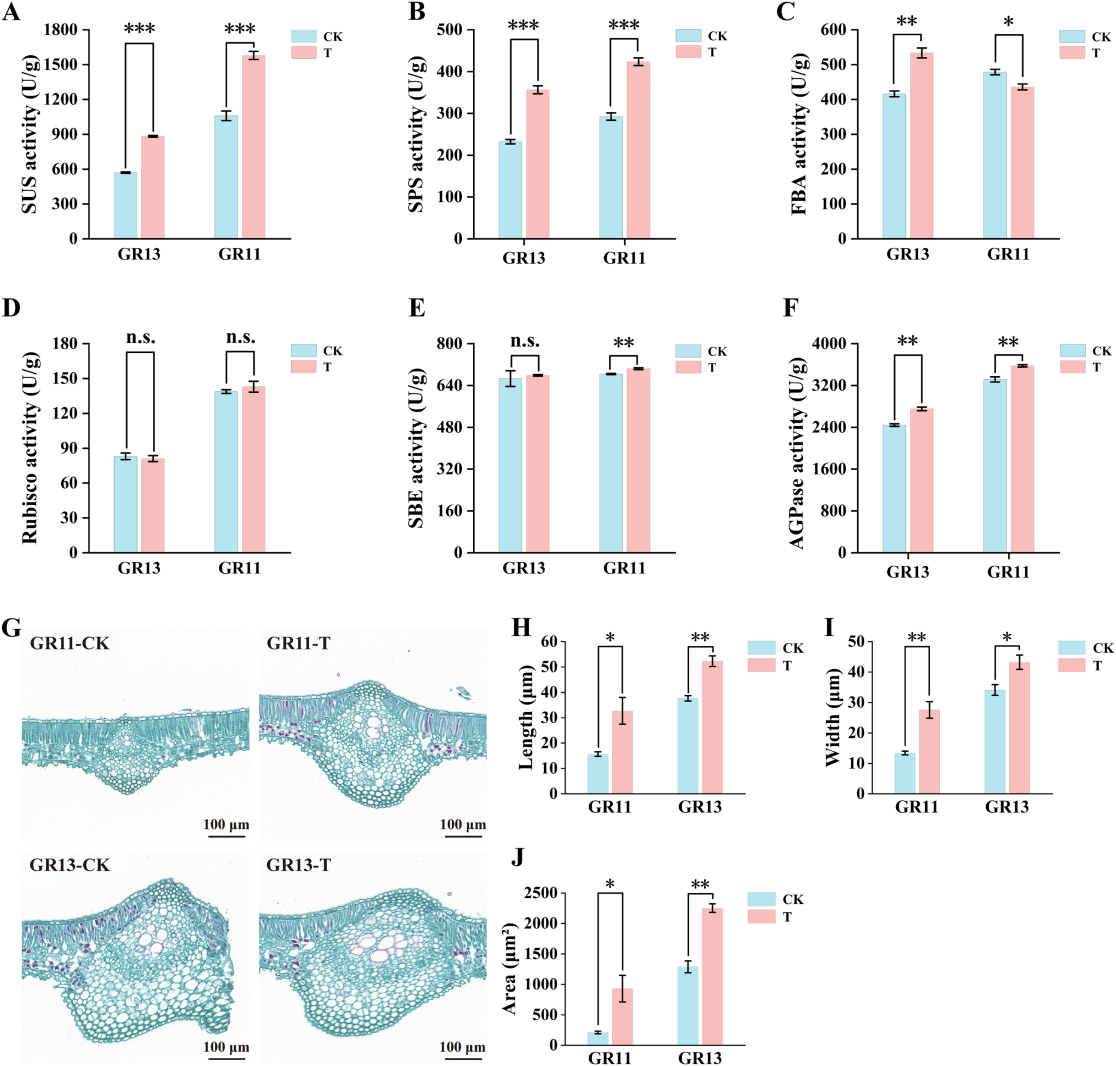
Effects of organic fertilizer treatment on starch synthesis-related enzyme activities and leaf anatomical structure in GR11 and GR13. Activities of SUS **(A)**, SPS **(B)**, FBA **(C)**, and Rubisco **(D)** in the leaves of GR11 and GR13; Activities of SBE **(E)** and AGPase **(F)** in their tuberous roots. **(G)** Paraffin section micrographs of leaves from GR11 and GR13 under different treatments. Quantitative analyses of xylem cell length **(H)**, width **(I)**, and area **(J)**. Values are presented as mean ± SE. Fisher’s LSD test was used for multiple comparisons, *p < 0.05, **p < 0.01, ***p < 0.001; n.s., no significant difference (*p* > 0.05).

At the mature stage of cassava, leaves and tuberous roots from two cassava varieties were collected separately. The activities of enzymes including SUS, SPS, FBA, and Rubisco in leaves, as well as SBE and AGPase in tuberous roots were determined (**Figures 1A–F**). The results showed that after the application of organic fertilizer, the activities of SUS and SPS in leaves, and SBE and AGPase in tuberous roots of GR11, were significantly higher than those in the control group, increasing by 49.13%, 44.79%, 3.00%, and 7.83%, respectively. In contrast, FBA activity in leaves was significantly lower than that in the control group, decreasing by 8.88%, while Rubisco activity showed no significant difference compared to the control. For GR13, organic fertilizer treatment significantly increased the activities of SUS, SPS, and FBA enzymes in leaves, as well as AGPase enzyme activity in roots, by 54.98%, 53.43%, 28.28%, and 12.71%, respectively, compared with the control group. Only the activities of leaf Rubisco and tuberous root SBE showed no significant differences from the control group. In addition, the response of FBA activity to fertilizer treatment varied among different varieties. In GR11, FBA activity decreased significantly under organic fertilizer treatment, whereas in GR13, FBA activity increased significantly. Overall, organic fertilizer treatment had a significant promoting effect on the activities of leaf SUS, SPS, and tuberous root AGPase in both cassava varieties.

### 3.3 Effects of organic fertilizer treatment on leaf structure of cassava

To further analyze the physiological basis of organic fertilizer in promoting cassava starch accumulation, the anatomical structure of the leaves of two cassava varieties at the mature stage was examined. The results showed that the vascular bundles in the leaves were fan-shaped, with clearly defined and distinguishable tissue boundaries, and the pith, xylem, and phloem were normally developed (**Figure 1G**). After organic fertilizer treatment, the length, width, and area of xylem cells in the leaves of both GR11 and GR13 were significantly greater than those in the control group (**Figures 1H–J**). Specifically, these parameters in GR11 were 2.08, 2.06, and 4.40 times those of the control, respectively, while in GR13, they were 1.39, 1.27, and 1.75 times those of the control, respectively.

### 3.4 Transcriptomic analysis of cassava following organic fertilizer treatment

#### 3.4.1 Data quality assessment

To eliminate interference from poor-quality data, raw reads from the 12 samples underwent qualitycontrol. After removing low-quality sequences, a total of 72.68 Gb of clean data were obtained. TheQ20 base ratio (bases with sequencing quality ≥ 20) ranged from 98.29% to 98.67%, the Q30 base ratio (bases with sequencing quality ≥ 30) ranged from 95.77% to 96.47%, and the GC content ranged from 42.48% to 43.56% (**Supplementary Table 2**). The mapping rate of clean reads to the cassava reference genome (Manihot_esculenta v8Ensembl 60 index) was between 97.95% and 98.31% (**Supplementary Table 3**), indicating high-quality sequencing data. Pearson correlation analysis was performed onsamples from different treatments (**Supplementary Figure 1**). The correlation coefficients between biological replicates were all above 0.953, indicating that the analyzed data are valid and reliable for further analysis.

#### 3.4.2 Statistical analysis of DEGs

**Figure 2:**
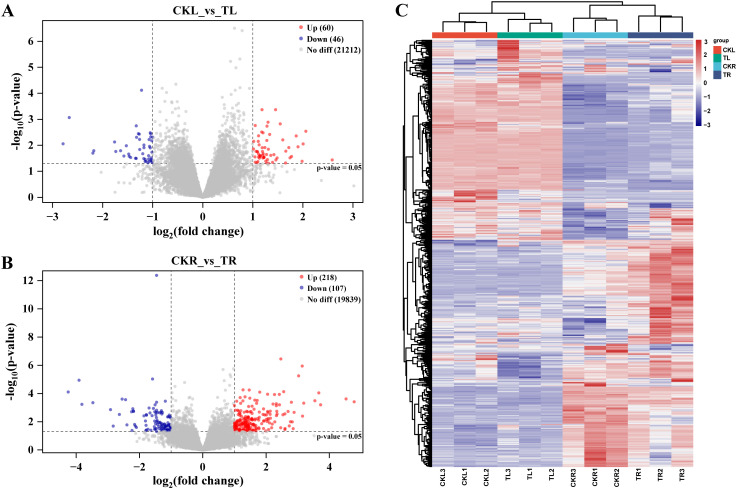
Volcano plots of DEGs in the CKL_vs_TL **(A)** and CKR_vs_TR **(B)** comparison groups, respectively. Each dot represents a gene; the x-axis shows log_2_(Fold Change), and the y-axis shows −log10(*p*-value). Red dots: up-regulated genes; blue dots: down-regulated genes; gray dots: genes with no significant difference. **(C)** Heatmap of DEGs cluster analysis. Each column represents a sample, and each row represents a gene. Color intensity reflects expression level: red for higher expression, blue for lower expression.

We performed quantitative statistical analysis and generated volcano plots for the DEGs of GR13 in two tissue-specific comparison groups: leaves (CKL_vs_TL) and tuberous roots (CKR_vs_TR) under different treatments (**Figures 2A, B**; **Supplementary Table 4**). The results indicated that the number of up-regulated genes exceeded that of down-regulated genes in both groups. Specifically, 106 DEGs were identified in the leaf group (60 up-regulated, 46 down-regulated), while 325 DEGs were detected in the tuberous root group (218 up-regulated, 107 down-regulated). Additionally, cluster analysis of samples and genes was performed based on the expression levels of all DEGs (**Figure 2C**). The results revealed distinct differences in gene expression profiles between treatmentgroups, with consistent expression trends observed among biological replicates. To further validatethe reliability of the transcriptome sequencing data, four genes were randomly selected from each of the two comparison groups for qRT-PCR verification. The results demonstrated that the up-regulated and down-regulated trends of gene expression detected by qRT-PCR were consistent with those observed in the transcriptome sequencing data (**Supplementary Figure 2**), confirming the reliability of the transcriptome sequencing results.

#### 3.4.3 GO enrichment analysis of DEGs

**Figure 3:**
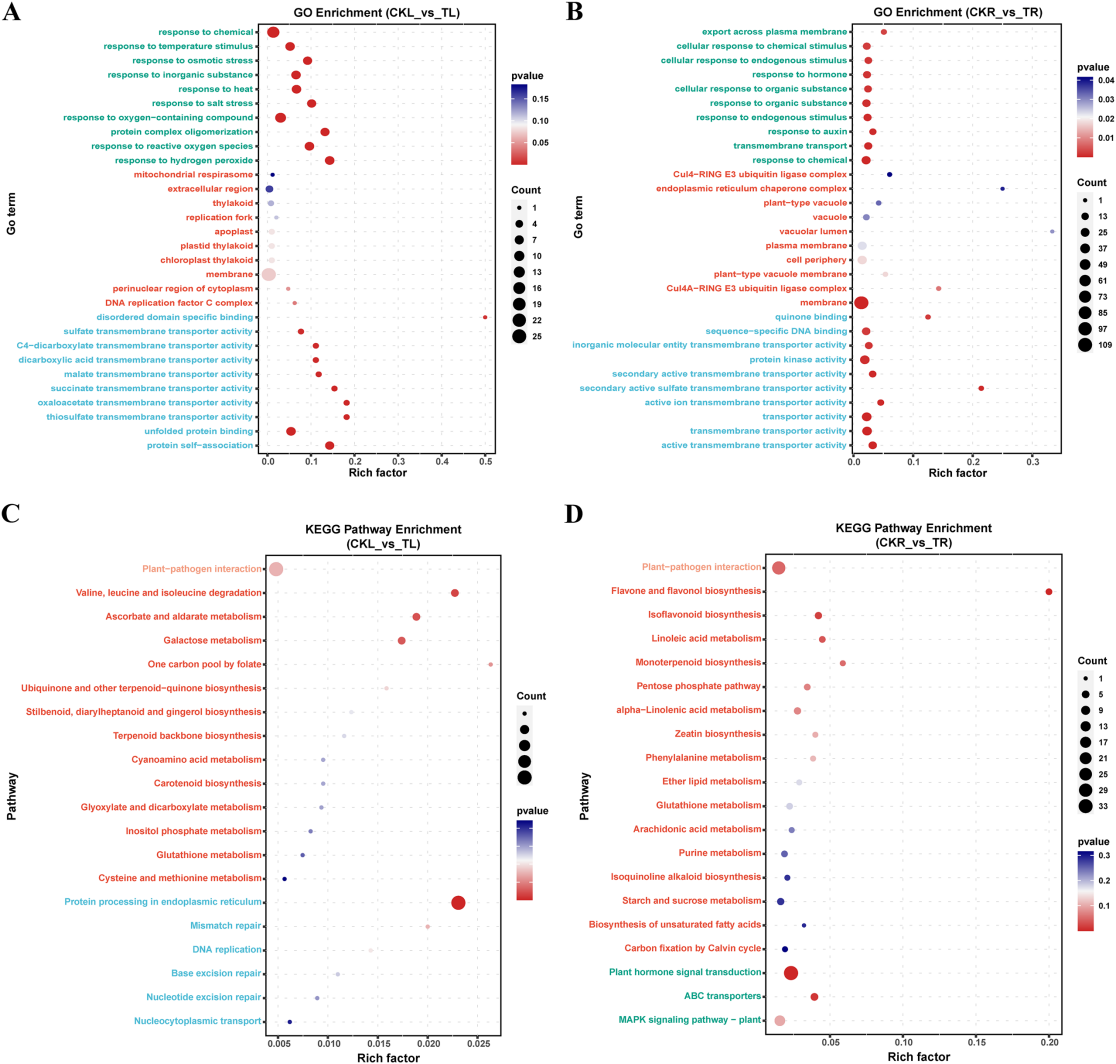
GO and KEGG enrichment bubble plots of DEGs. **(A, B)** GO enrichment plots: The x-axis represents the rich factor (the ratio of DEGs to total genes in each GO term), and the y-axis displays GO term names. Dot color indicates the enrichment *p*-value, dot size corresponds to the DEG count, and text color denotes the functional category (green: BP, red: CC, blue: MF). **(C, D)** KEGG enrichment plots: The x-axis represents the rich factor (the ratio of DEGs to total genes in each pathway), and the y-axis displays KEGG pathway names. Dot color indicates the enrichment *p*-value, dot size corresponds to the DEG count, and text color denotes the functional category (orange: organismal systems, red: metabolism, blue: genetic information processing, and green: environmental information processing).

GO functional annotation and enrichment analyses were conducted on DEGs of each comparison group under different treatments. The results were categorized into three groups: biological process (BP), cellular component (CC), and molecular function (MF). The top 10 most significant GO terms in each category were visualized (**Figures 3A, B**). In the leaf comparison group (CKL_vs_TL), BP enrichment was predominantly associated with stress-responsive terms, including response to hydrogen peroxide, response to reactive oxygen species, and response to salt stress, with 8, 8, and 7 DEGs, respectively. For CC, membrane, DNA replication factor C complex, and perinuclear region of cytoplasm were notably enriched, containing 26, 1, and 1 DEGs, respectively. MF enrichment primarily involved substance transport-related activities, including succinate transmembrane transporter activity, oxaloacetate transmembrane transporter activity, and thiosulfate transmembrane transporter activity, each comprising 2 DEGs. These results suggest that DEGs in CKL_vs_TL are mainly involved in responses to external stimuli, cellular structure formation, and transmembrane substance transport (**Figure 3A**, **Supplementary Figure 3A**). In the tuberous root comparison group (CKR_vs_TR), BP enrichment was dominated by highly significant terms, including export across plasma membrane, response to auxin, cellular response to endogenous stimulus, and response to hormone, with 5, 11, 14, and 17 corresponding DEGs, respectively. For CC, membrane showed the most significant enrichment, involving 115 DEGs. MF enrichment focused on transporter activity-related functions, such as secondary active sulfate transmembrane transporter activity, active ion transmembrane transporter activity, and active transmembrane transporter activity, with 3, 9, and 21 DEGs, respectively. These results suggest that DEGs expression differences in CKR_vs_TR are primarily involved in endogenous signal response, membrane structure, and transmembrane substance transport (**Figure 3B**; **Supplementary Figure 3B**).

#### 3.4.4 KEGG pathway analysis of DEGs

KEGG pathway enrichment analysis was conducted on the DEGs of each comparison group under various treatments. The top 20 pathways with the highest enrichment significance in each group were selected for visualization (**Figures 3C, D**). In the leaf comparison group (CKL_vs_TL), DEGs were significantly enriched in pathways including protein processing in endoplasmic reticulum, one carbon pool by folate, ascorbate and aldarate metabolism, galactose metabolism, valine, leucine, and isoleucine degradation, and plant-pathogen interaction (**Figure 3C**), corresponding to 9, 1, 2, 2, 2, and 9 DEGs, respectively (**Supplementary Figure 4A**). In the tuberous root comparison group (CKR_vs_TR), DEGs were significantly enriched in pathways such as flavone and flavonol biosynthesis, ABC transporters, plant hormone signal transduction, isoflavonoid biosynthesis, linoleic acid metabolism, and plant-pathogen interaction (**Figure 3D**), with 3, 5, 34, 4, 4, and 26 DEGs, respectively (**Supplementary Figure 4B**).

### 3.5 Metabolomic analysis of cassava following organic fertilizer treatment

#### 3.5.1 Data quality assessment

**Figure 4:**
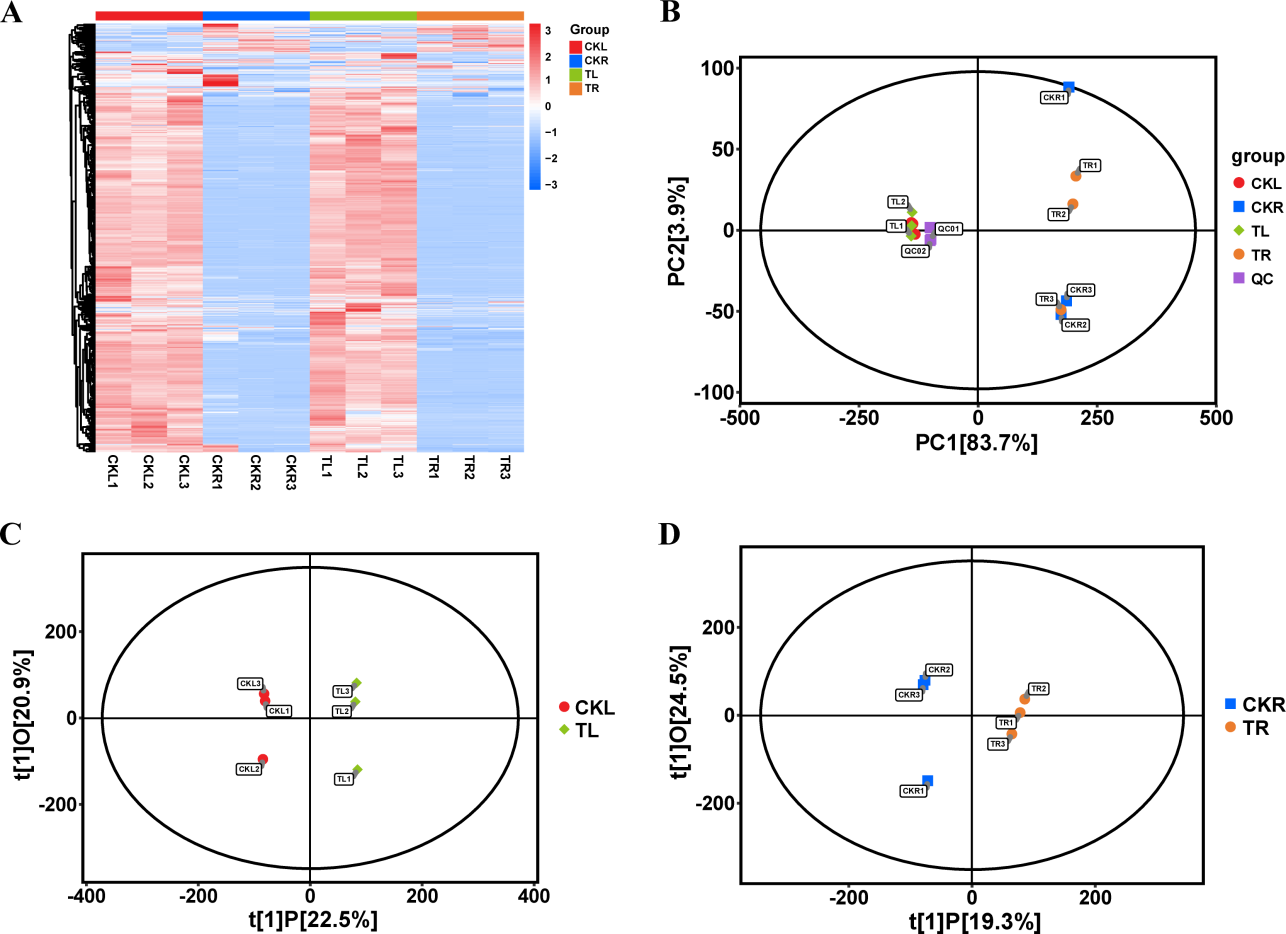
Quality assessment of metabolomics data. **(A)** Hierarchical clustering analysis (HCA) heatmap of all samples. The x-axis represents samples, the y-axis represents metabolites, and colors indicate relative metabolite abundance (red: high, blue: low); **(B)** PCA score plot of all samples, including QC samples. The x-axis (PC1) and y-axis (PC2) represent the scores of the first and sec-ond principal components, respectively. Each scatter represents one sample, with colors and shapes indicating different groups; OPLS-DA score plots for CKL_vs_TL **(C)** and CKR_vs_TR **(D)**, respectively. The x-axis [t[1]P] and y-axis (t[1]O) represent predictive and orthogonal principal components, with scatter colors and shapes corresponding to experimental groups.

After data normalization, cluster heatmap analysis was conducted on all samples (**Figure 4A**). The results revealed significant differences in metabolite content among groups, with consistent biological replicates within each group. Principal component analysis (PCA) demonstrated strong intra-group clustering, confirming high consistency in metabolite composition and reliable experimental reproducibility. Additionally, the percentages of variance explained by PC1 and PC2 are 83.7% and 3.9%, respectively, leaf and tuberous root samples were distinctly separated along PC1, indicating tissue-specific metabolite profiles (**Figure 4B**). Further orthogonal partial least squares discriminant analysis (OPLS-DA) showed clear inter-group separation and tight intra-group clustering in both comparison sets (**Figures 4C, D**). In summary, the comparison groups exhibited significant metabolite differences and robust intra-group reproducibility, providing a reliable foundation for subsequent analyses.

#### 3.5.2 Statistical analysis of DEMs

DEMs were identified using the criteria of VIP > 1.0 and *p* < 0.05, followed by statistical analysis (**Table 2**; **Supplementary Table 5**). The results showed that a total of 1, 934 metabolites were detected across both comparison groups, with the number of up-regulated metabolites exceeding that of down-regulated metabolites in each group. Specifically, 109 DEMs were identified in the CKL_vs_TL group, including 66 up-regulated and 43 down-regulated metabolites, while 68 DEMs were identified in the CKR_vs_TR group, comprising 40 up-regulated and 28 down-regulated metabolites (**Table 2**).

**Table 2:** Statistical results of DEMs.

Group	Total detected metabolites	Total DEMs	Up-regulated	Down-regulated
CKL_vs_TL	1934	109	66	43
CKR_vs_TR	1934	68	40	28

#### 3.5.3 KEGG enrichment analysis of DEMs

**Figure 5:**
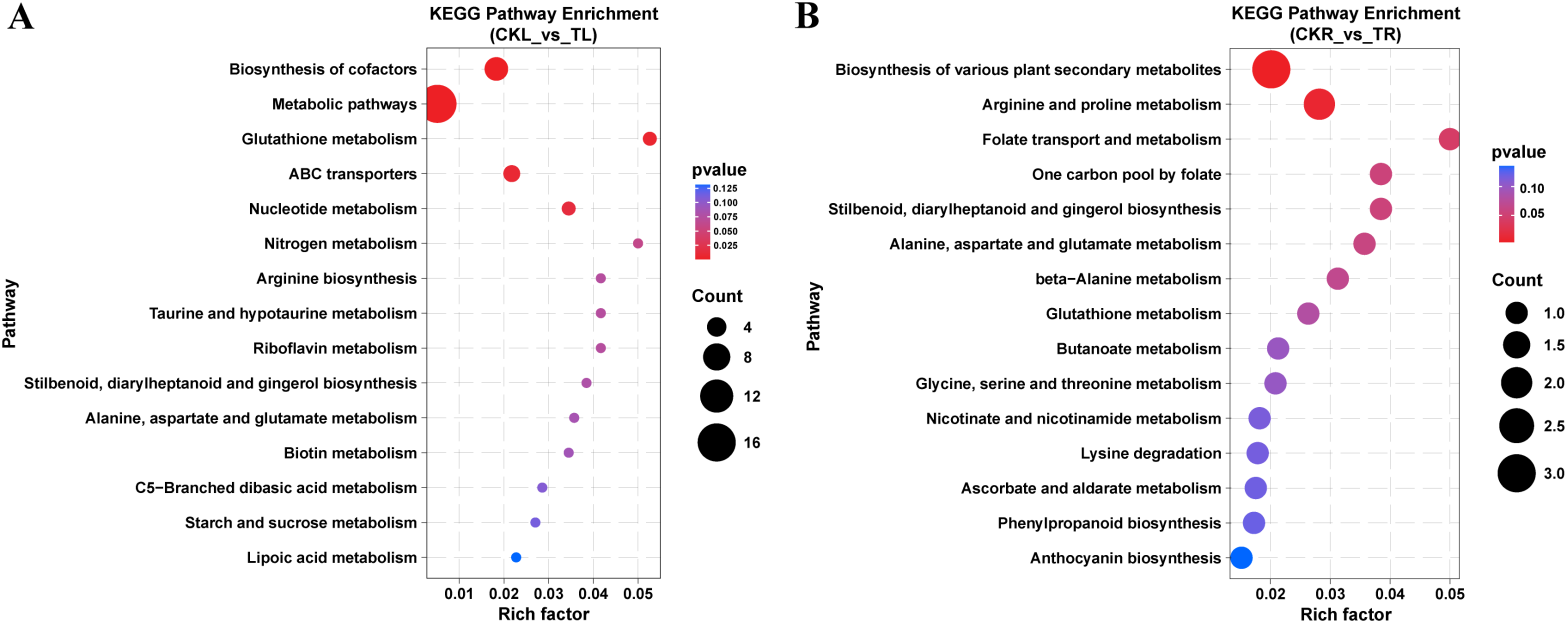
KEGG enrichment bubble plots of DEMs. The x-axis represents the rich factor for each pathway, while the y-axis displays the names of the KEGG metabolic pathways. The size of the dots indicates the number of DEMs enriched in each corresponding pathway, and the color of the dots reflects the *p*-value. **(A)** CKL_vs_TL group; **(B)** CKR_vs_TR group.

KEGG enrichment analysis was conducted on the DEMs of each comparison group under various treatments. The results showed that DEMs in the leaf comparison group (CKL_vs_TL) were significantly enriched in pathways including glutathione metabolism, nucleotide metabolism, ABC transporters, biosynthesis of cofactors, and metabolic pathways (**Figure 5A**). In contrast, DEMs in the tuberous root comparison group (CKR_vs_TR) were significantly enriched in pathways such as biosynthesis of various plant secondary metabolites, arginine and proline metabolism, folate transport and metabolism, one carbon pool by folate, and stilbenoid, diarylheptanoid, and gingerol biosynthesis (**Figure 5B**).

Based on the KEGG enrichment analysis of DEMs, a comprehensive pathway analysis was conducted incombination with topological analysis (**Supplementary Figure 5**). The results showed that DEMs in the leaf comparison group (CKL_vs_TL) were primarilyenriched in pathways including glutathione metabolism, riboflavin metabolism, alanine, aspartate andglutamate metabolism, starch and sucrose metabolism, and arginine and proline metabolism (**Supplementary Figure 5A**). In contrast, DEMs in the tuberous root comparison group (CKR_vs_TR) were mainly enrichedin pathways such as stilbenoid, diarylheptanoid and biosynthesis, ascorbate and aldarate metabolism,and glutathione metabolism (**Supplementary Figure 5B**).

### 3.6 Integrated analysis of transcriptome and metabolome

**Figure 6:**
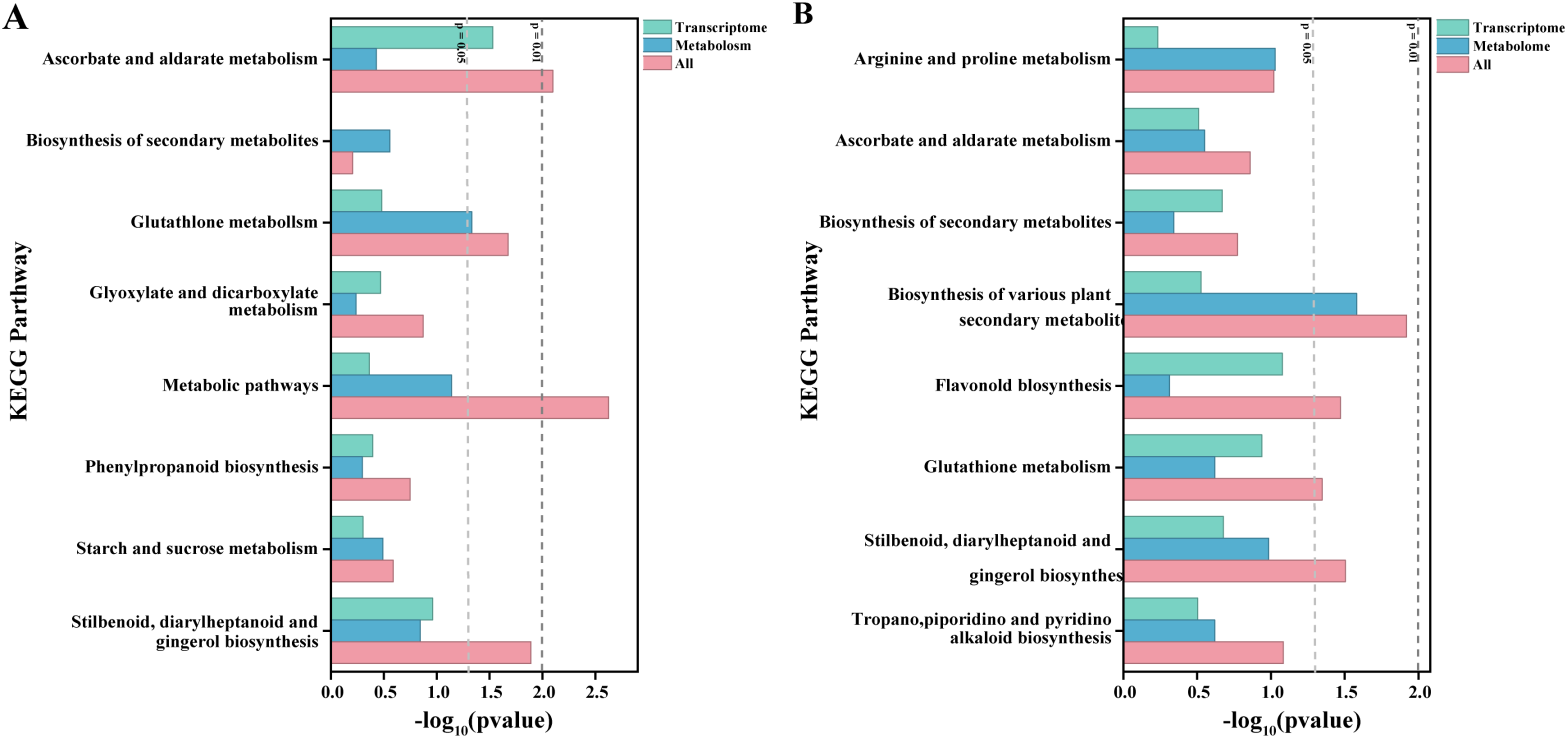
Integrated transcriptome-metabolome KEGG enrichment bar plots for CKL_vs_TL **(A)** and CKR_vs_TR **(B)**, respectively. The x-axis represents the −log10(*p*-value); the y-axis represents pathways. Different colors indicate different omics layers. The left light-colored dashed line denotes *p* = 0.05, and the right dark-colored solid line denotes *p* = 0.01.

To clarify the association between DEGs and DEMs, an integrated analysis of transcriptomic and metabolomic data was performed in this study (**Figure 6**). The results showed that both the glutathione metabolism pathway (map00480) and stilbenoid, diarylheptanoid and gingerol biosynthesis pathway (map00945) were significantly enriched in both the CKL_vs_TL group and CKR_vs_TR group. Additionally, the starch and sucrose metabolism pathway (map00500) was specifically enriched in the leaf group (**Figure 6A**).

**Figure 7:**
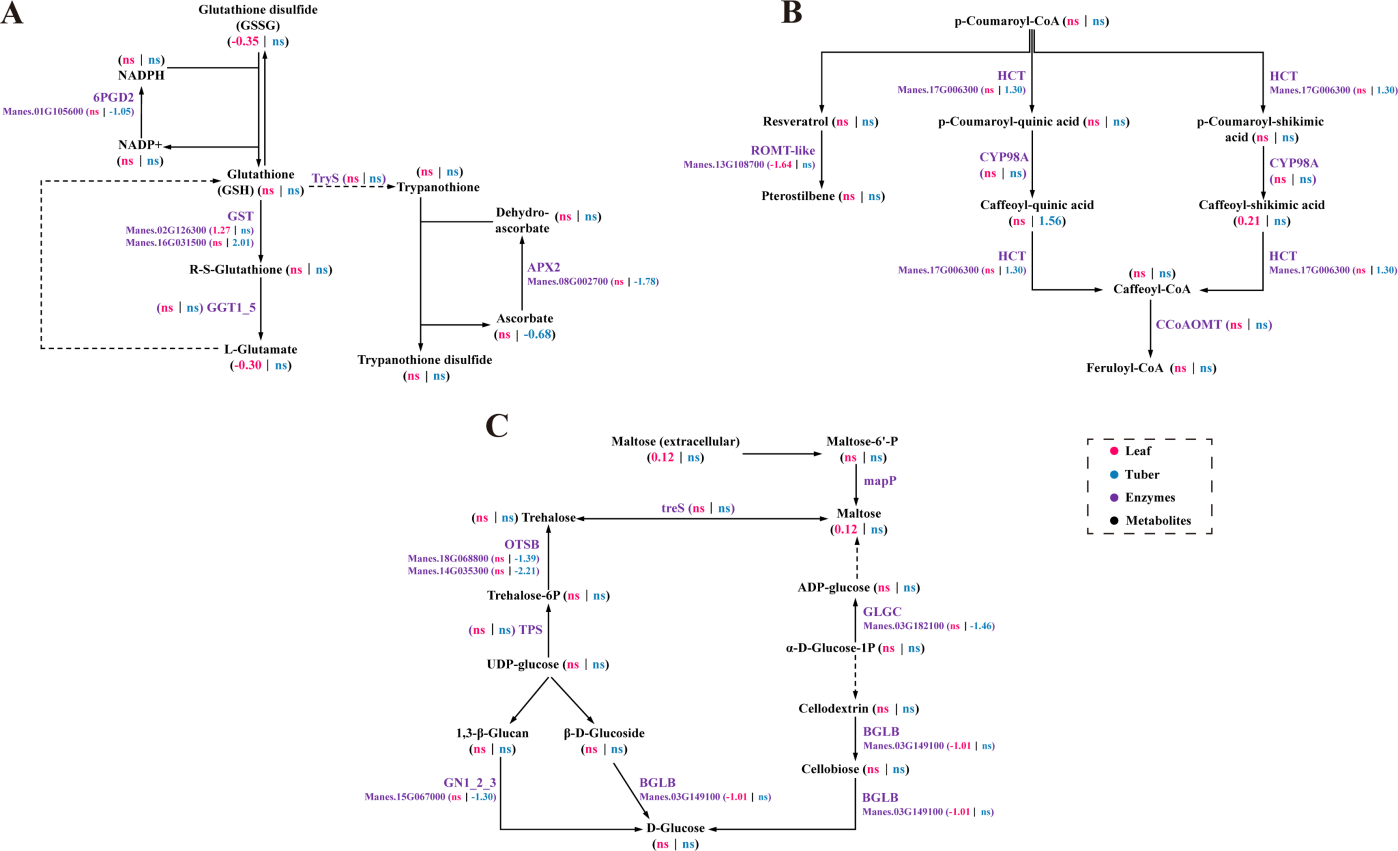
Pathway networks of glutathione metabolism **(A)**, stilbenoid-diarylheptanoid-gingerol biosynthesis **(B)**, and starch-sucrose metabolism **(C)** in cassava, respectively. Nodes represent metabolites (black font), while enzymes are labeled on the connecting lines (purple font). Red and blue numbers indicate the log_2_(Fold Change) of enzymes or metabolites in CKL_vs_TL and CKR_vs_TR, respectively; ns, no significant difference. Substances unaffected by organic fertilizer application are omitted in from figure and connected by dashed lines.

In the glutathione metabolism pathway (map00480, **Figure 7A**), the levels of glutathione disulfide (GSSG) and L-glutamate in cassava leaves under organic fertilizer application were significantly decreased, while the expression of the glutathione S-transferase gene *MeGST-1* (Manes.02G126300) was significantly up-regulated. In contrast, in cassava tuberous roots, ascorbate levels were significantly down-regulated; the expression of another glutathione S-transferase gene, *MeGST-2* (Manes.16G031500), was significantly up-regulated, whereas the expression levels of *MeApx2* (Manes.08G002700, encoding L-ascorbate peroxidase 2) and *Me6pgd2* (Manes.01G105600, encoding 6-phosphogluconate dehydrogenase 2, decarboxylating) were both significantly down-regulated.

In the stilbenoid, diarylheptanoid, and gingerol biosynthesis pathway (map00945, **Figure 7B**), the content of caffeoyl-shikimic acid was significantly up-regulated in cassava leaves following organic fertilizer application, whereas the expression of *MeROMT-like* (Manes.13G108700), which encodes a trans-resveratrol di-O-methyltransferase-like protein, was significantly downregulated. Conversely, both the levels of caffeoyl-quinic acid and the expression of *MeHct* (Manes.17G006300), encoding shikimate O-hydroxycinnamoyltransferase, were significantly up-regulated in cassava tuberous roots.

In the starch and sucrose metabolism pathway (map00500, **Figure 7C**), maltose levels were significantly increased in cassava leaves under organic fertilizer application, while the expression of *MebglB* (Manes.03G149100), which encodes β-glucosidase, was significantly decreased. In contrast, the expression levels of four genes were significantly down-regulated in cassava tuberous roots: *MeglgC* (Manes.03G182100, encoding glucose-1-phosphate adenylyltransferase), *MeGN1_2_3* (Manes.15G067000, encoding endo-1, 3-β-glucanase), and *MeOTSB-A* (Manes.14G035300) and *MeOTSB-D* (Manes.18G068800), both encoding trehalose-6-phosphate phosphatase.

### 3.7 O2PLS model and correlation analysis

**Figure 8:**
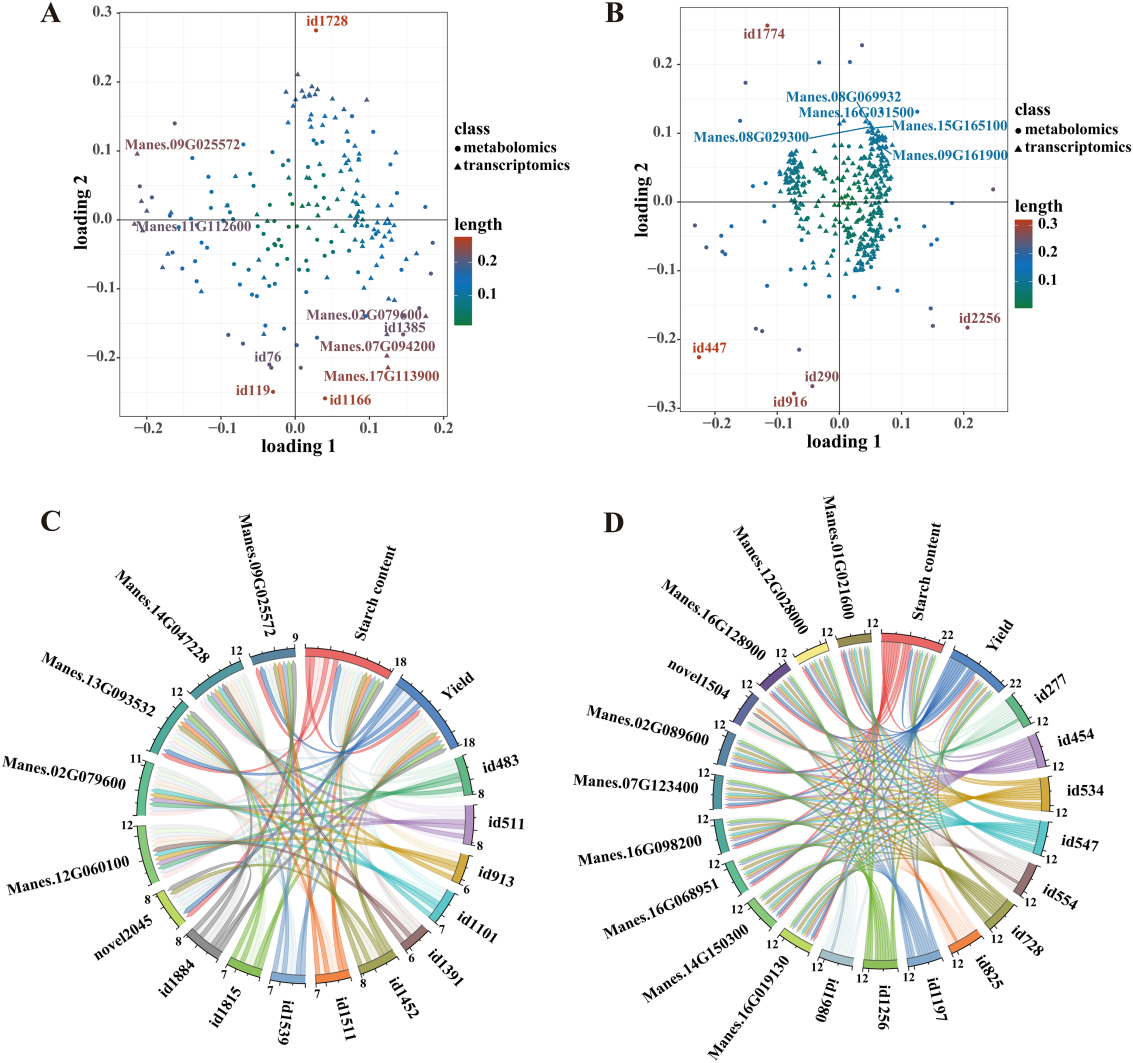
Multi-omics association analysis of starch synthesis and yield regulation in cassava. **(A, B)** Two-way orthogonal partial least squares (O2PLS) loading plots. Circles represent differential metabolites, triangles represent differentially expressed genes, and color intensity indicates the contribution of each metabolite or gene to the model. Core candidate genes and metabolites associated with starch synthesis and yield are highlighted. **(C, D)** Correlation analysis and chord diagrams of agronomic traits, differentially expressed genes, and differential metabolites. Connecting bands indicate significant correlations (*p<* 0.05), with dark bands representing positive correlations and light bands representing negative correlations.

An integrated analysis of differentially expressed genes and differential metabolites was conducted using the O2PLS model (**Figures 8A, B**). Based on absolute loading values, the top five differential genes with the greatest influence in leaves were Manes.09G025572, Manes.11G112600, Manes.02G079600, Manes.07G094200, and Manes.17G113900. The top five differential metabolites in leaves were id1728, id76, id119, id1166, and id1385. In storage roots, the top five differential genes with the greatest influence were Manes.08G069932, Manes.16G031500, Manes.08G029300, Manes.09G161900, and Manes.15G165100, while the top five differential metabolites were id1774, id447, id2256, id290, and id916.

To further identify regulatory modules exhibiting coordinated changes with agronomic traits, Pearson correlation analysis was conducted to examine the relationships among starch content, yield, differentially expressed genes, and differential metabolites (**Figures 8C, D**). Gene sets with similar expression patterns and significant correlations with traits(*p* < 0.05) were defined as potential regulatory modules. The resultsshowed that in leaves, both starch content and yield were significantly positively correlated with gene set I (novel2045, Manes.13G093532, Manes.09G025572), but significantly negatively correlated with gene set II (Manes.12G060100, Manes.02G079600, Manes.14G047228). Metabolite module I (id483, id511, id913, id1101, id1391) and metabolite module II (id452, id1511, id1539, id1815, id1884) in leaves were also significantly associated with the two aforementioned gene sets, respectively. In storage roots, gene set III (Manes.16G019300, Manes.14G150300, Manes.16G068951, Manes.16G098200, Manes.07G123400, Manes.02G089600, Manes.16G128900, Manes.12G028000, Manes.01G021600) was not only significantly positively correlated with starch content and yield but also significantly positively correlated with metabolite module III (id454, id534, id547, id728, id1197, id1256). Detailed annotations of these genes and metabolites are provided in **Supplementary Table 6**.

## 4 Discussion

### 4.1 Effects of organic fertilizer on the starch biosynthesis pathway in cassava

Rational fertilization is a fundamental agronomic practice for improving crop yield and quality. However, numerous studies have shown that excessive use of chemical fertilizers can lead to a decline in both yield and quality. Compared to chemical fertilizers, organic fertilizers offer significant advantages in cultivating high-quality, high-yield crops (Abd El-Azeim et al., 2020; Shi et al., 2023). As a typical tropical tuber crop, cassava exhibits distinct biological characteristics compared to cereals and other food crops. First, the photosynthetic products from cassava leaves are not stored in seeds but are transported over long distances to the underground tubers for starch synthesis and storage. Therefore, root development and starch accumulation efficiency directly determine its economic value (Mehdi et al., 2019). Second, the effects of chemical fertilizers are typically transient and fail to meet the prolonged growth cycle of cassava, which requires a sustained and stable nutrient supply (Adiele et al., 2021). Among commonly used organic fertilizers, chicken manure has been found to effectively increase both fresh tuber and starch yields of cassava grown in low-yield sandy soils (Jindaluang et al., 2025). In this study, compared with the sole application of chemical fertilizer, treatment with chicken manure significantly increased the tuber yields and starch content of GR11 and GR13. Specifically, tuber yields increased by 13.72% and 26.08%, respectively, while starch content increased by 11.21% and 9.90%, respectively (**Table 1**). As a high-quality organic fertilizer, chicken manure gradually and continuously releases essential nutrients such as nitrogen, phosphorus, and potassium, providing a stable nutrient supply that supports the ongoing enlargement of storage roots. Additionally, the organic matter it contains effectively improves the physical and chemical properties of the soil, alleviates soil compaction, and enhances soil aeration. These benefits promote root growth and create an optimal soil environment for cassava root expansion and starch accumulation. These findings further confirm the value of organic fertilizer in the high-quality, high-yield cultivation of cassava.

As the primary organ of photosynthesis and the main site of assimilate synthesis, the metabolicactivity of leaves directly determines the carbon supply capacity at the source and plays a crucialregulatory role in starch synthesis and accumulation (Tappiban et al., 2019; Liu et al., 2026). Starch is generally classified into two types: transitory starch and storage starch. Transitory starch, synthesized by photosynthesis in leaves during the day, is degraded at night into maltose, glucose, and other products. These products are then converted into sucrose through cytoplasmic metabolism, providing a carbon source for plant growth and assimilate transport (Pfister and Zeeman, 2016; Smith and Zeeman, 2020). Studies have shown that maltose accumulation may help protect the photosynthetic electron transport chain, as well as proteins and membranes within the chloroplast (Kaplan and Guy, 2004). In this study, the expression levels of multiple members of the α-amylase (AMY) and β-amylase (BAM) gene families were generally upregulated in the TL treatment group, indicating that the degradation efficiency of transient starch in leaves was significantly higher in the TL group than in the control group (CKL) (**Supplementary Figure 6**). Additionally, organic fertilizer (TL) treatment significantly increased maltose content in cassava leaves (**Figure 7C**). These results suggest that organic fertilizer (TL) treatment may accelerate transient starch degradation by promoting the expression of *AMY* and *BAM* genes in leaves, which could further facilitate the transport of photosynthetic products (e.g., maltose) to underground tubers. This process may improve carbon source utilization efficiency in leaves, reduce ineffective retention of carbon sources, and provide a reliable carbon supply for starch accumulation in tubers.

The leaf structure underpins its physiological functions. Treatment with organic fertilizer significantly optimized the xylem structure of cassava leaves by increasing the xylem cell area (**Figure 1**). This structural modification could enhance the transport efficiency of water and mineral nutrients within the leaves, thereby ensuring the stable and efficient progression of photosynthesis (Kreuzwieser et al., 2002; Secchi and Zwieniecki, 2016). Additionally, the study found that the increase in xylem cell area in GR13 under fertilization treatment was smaller than that in GR11. This may be because the xylem cell area of GR13 was already significantly larger than that of GR11 in the absence of organic fertilizer, leaving relatively limited room for further enlargement. These results indicate, to some extent, that xylem cell characteristics in cassava leaves are jointly regulated by both genotype and fertilization treatment. Regarding the activities of enzymes related to starch synthesis, the results showed that organic fertilizer treatment significantly increased the activities of SPS and SUS in cassava leaves (**Figure 1**). SPS and SUS are key enzymes involved in the conversion of photosynthetic products into sucrose, and their enhanced activity is conducive to promoting sucrose synthesis, thus providing a material basis for carbon export in the plant (Volpicella et al., 2016; Bharali and Baruah, 2022).

As the primary sink organ for starch storage in cassava, starch biosynthetic capacity in storage roots is a key determinant of the overall efficiency of carbon accumulation. AGPase, a crucial rate-limiting enzyme in the starch biosynthesis pathway, exhibits a significant positive correlation between its activity and starch biosynthetic capacity (Tang et al., 2016). In this study, the application of organic fertilizer significantly increased AGPase activity in cassava storage roots (**Figure 1**), thereby enhancing the starch biosynthetic potential at the sink. This may be because organic fertilizer, by optimizing leaf structure, enhancing photosynthesis, and promoting sucrose synthesis in leaves and its transport to tubers, provides a more abundant substrate for starch synthesis in tubers, thereby up-regulating AGPase activity and ultimately achieving a significant increase in both yield and starch content. In contrast, as a starch branching enzyme, SBE primarily participates in amylopectin synthesis by catalyzing the formation of branch structures within starch molecules and has a relatively limited effect on total starch content. Previous studies have demonstrated that cassava SBE exhibits distinct spatiotemporal expression patterns (Pei et al., 2015), with significantly higher expression in stems and roots during the late developmental stages of cassava, and its expression is influenced by genotype (Salehuzzaman et al., 1992; Baguma et al., 2003). The present study found that SBE activity in GR11 was higher than in GR13; under organic fertilizer treatment, SBE activity in GR11 increased significantly, whereas no significant change was observed in GR13. These findings indicate that GR11 may have a greater capacity to finely regulate starch structure and is more responsive to fertilization treatment. FBA is a key enzyme involved in both the Calvin cycle and the glycolytic pathway. Research by Stitt et al. (1991) found that increased carbohydrate content in plants leads to reduced levels of enzymes related to the Calvin cycle. This study demonstrates that under organic fertilizer treatment, FBA activity in GR11 leaves significantly decreased, while SUS and SPS activities markedly increased, suggesting that enhanced sucrose synthesis capacity may inhibit FBA activity through a negative feedback mechanism. In contrast, SUS, SPS, and FBA activities all increased significantly in GR13 leaves, indicating a distinct carbon allocation regulatory pattern under organic fertilizer treatment compared to GR11. The differing carbon metabolism response patterns between these varieties may be a major factor contributing to the variations in yield and starch content between GR11 and GR13.

### 4.2 Effects of organic fertilizer on the antioxidant pathway in cassava

In China, cassava is typically cultivated on barren, sloping lands in the southern regions, where drought and nutrient deficiencies are the primary abiotic stresses that reduce cassava yield. Therefore, enhancing stress tolerance in cassava has become a crucial focus of research for its improvement. In the natural growth of plants, there is an inherent “growth-defense trade-off” in carbon resource allocation. When carbon is preferentially directed toward growth processes such as starch accumulation and biomass increase, its allocation to defense processes, including the synthesis of immune substances and stress responses, is correspondingly reduced, and vice versa (He et al., 2022). In this study, organic fertilizer treatment was found to significantly increase cassava yield and starch content, while also regulating the metabolism of stress-related compounds.

β-1, 3-glucan is predominantly present in plants in the form of callose, a polysaccharide essential for cell wall assembly and structural integrity (Rebaque et al., 2021; Li et al., 2023; Liu et al., 2023). β-1, 3-glucanase is a key hydrolytic enzyme that specifically catalyzes the breakdown of β-1, 3-glucan linkages. Accumulating evidence demonstrates that β-1, 3-glucanase plays a central role in cell wall remodeling and dynamic glucan homeostasis across diverse plant species (Gu et al., 2024). In this study, organic fertilizer application significantly downregulated the expression of the β-1, 3-glucanase gene *MeGN1_2_3* in cassava tuberous roots (**Figure 7C**). The reduced transcript abundance of this hydrolase suggests a diminished capacity for β-1, 3-glucan degradation. Collectively, these findings indicate that organic fertilizer may modulate cell wall remodeling processes in cassava tuberous roots by inhibiting β-1, 3-glucan hydrolysis.Following the application of organic fertilizer, the content of caffeoylshikimic acid in cassava leaves, caffeoylquinic acid in storage roots, and the expression level of *MeHCT* (the gene encoding shikimate hydroxycinnamoyl transferase) all increased significantly (**Figure 7B**). These phenolic secondary metabolites are key substances involved in plant stress resistance and defense (Claude et al., 2021), and their synthesis, like that of starch, depends on up-stream carbon sources derived from photosynthetic carbon assimilation (Thokchom et al., 2023). The simultaneous accumulation of these phenolic metabolites and starch is likely due to enhanced synthesis and translocation efficiency of plant carbon assimilates induced by organic fertilizer application, which increases the activities of SPS and SUS. This leads to a significant rise in the total amount of photosynthetic carbon assimilation, thereby providing sufficient carbon sources for both starch and phenolic secondary metabolite synthesis.

Furthermore, the application of organic fertilizer significantly upregulated the *MeGST* gene, which is crucial for glutathione biosynthesis, in cassava leaves and storage roots (**Figure 7A**). The glutathione metabolic pathway is a core mechanism by which plants maintain cellular redox homeostasis and resist abiotic stress (Noctor et al., 2024; Bose Mazumdar and Chattopadhyay, 2025). In leaves, the application of organic fertilizer significantly decreased GSSG and L-glutamate contents while markedly upregulating the expression of *MeGST-1* (Manes.02G126300), a gene encoding GST (**Figure 7A**). As the oxidized form of glutathione, the reduction of GSSG indicates diminished accumulation of oxidized glutathione and improved cellular redox homeostasis in leaves. L-glutamate, a precursor in glutathione biosynthesis, exhibited reduced levels not due to a deficiency in carbon supply. Instead, the application of organic fertilizer upregulated *MeGST* expression, accelerating the conversion of L-glutamate to glutathione and promoting the reductive regeneration of GSSG. This process mitigates oxidative damage and enhances glutathione utilization efficiency.

In summary, this study systematically elucidated the molecular mechanisms underlying the regulation of starch synthesis and stress tolerance in cassava by organic fertilizer at the multi-omics level. However, several limitations remain to be addressed. First, the study employed an equal application rate design (50 g per plant) rather than an equivalent NPK nutrient comparison. Essentially, organic matter was used to replace part of the nitrogen, phosphorus, and potassium nutrients, aiming to investigate the comprehensive effects of “organic matter plus low nutrient input” on crop growth under conventional field application rates. Future research will include an equivalent nutrient control treatment, in which single-element fertilizers will be applied to balance the NPK levels between organic fertilizer and chemical fertilizer treatments, thereby further clarifying the intrinsic biological regulatory effects of organic fertilizer. Second, although multiple types of organic fertilizers were evaluated, the field trials in this study encompassed a limited range of agroecological zones and cassava varieties; broader testing across diverse environments and genotypes is necessary to confirm the generalizability and stability of the observed regulatory mechanisms. Third, the significant enrichment of stress resistance-related pathways in the transcriptome suggests that future studies should conduct a combined analysis with physiological indicators, such as reactive oxygen species (ROS) content and the activities of antioxidant enzymes (SOD, POD, and CAT). Addressing these research gaps will enhance our understanding of the underlying mechanisms and support the development of precision organic strategies for sustainable, high-yield cassava production.

## 5 Conclusion

In this study, the regulatory mechanism of organic fertilizer on starch synthesis during cassava maturation was investigated through the combined analysis of agronomic trait determination, enzyme activity assays, leaf anatomical observations, and multi-omics approaches. The results demonstrated that organic fertilizer increased the area of xylem cells in leaves, the activity of key enzymes involved in starch synthesis (SUS, SPS, AGPase), and regulated multiple metabolic pathways including glutathione metabolism. These effects collectively contribute to simultaneous increases in storage root yield and starch content. The results of this study provide both a theoretical foundation and practical guidance for the rational application of organic fertilizer in high-quality, high-yield cassava cultivation, as well as insights into the physiological regulatory mechanisms of starch biosynthesis.

## Data Availability

The datasets presented in this study can be found in online repositories. The names of the repository/repositories and accession number(s) can be found in the article/**Supplementary Material**.
