# Supplementary material for: Integrated omics analysis on organic fertilizer-mediated regulation of starch synthesis in cassava
Source: Front Plant Sci. 2026 Jun 1;17:1821836. doi: 10.3389/fpls.2026.1821836 (PMC13265497; doi:10.3389/fpls.2026.1821836)
Supplement: Supplementary file 1 [file Table1.docx]

Supplementary Material

# Supplementary Figures


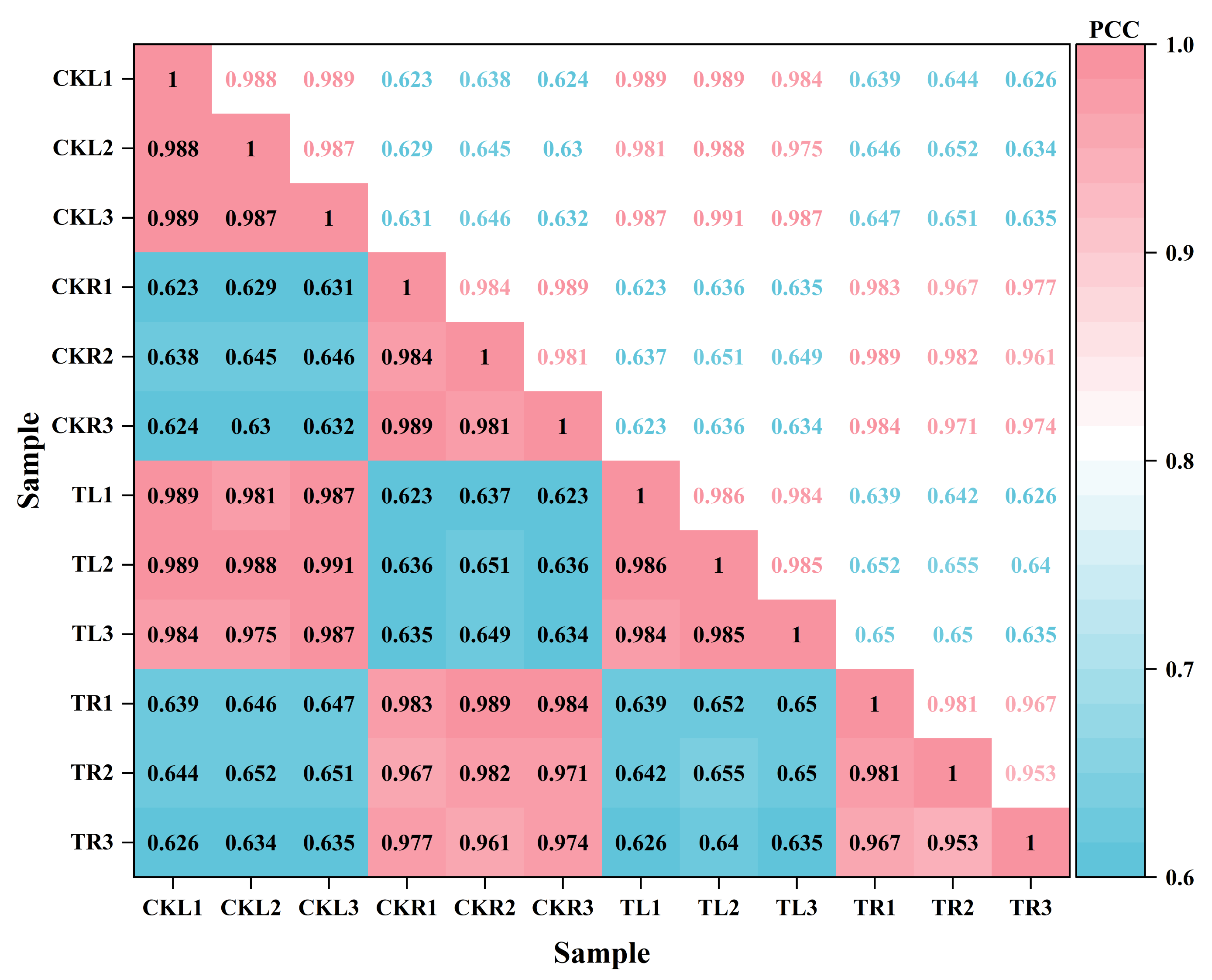


**Supplementary Figure 1.** Correlation analysis of transcriptome samples. The abscissa and ordinate represent individual samples, respectively, while the color intensity indicates the magnitude of the correlation coefficient between the two samples.


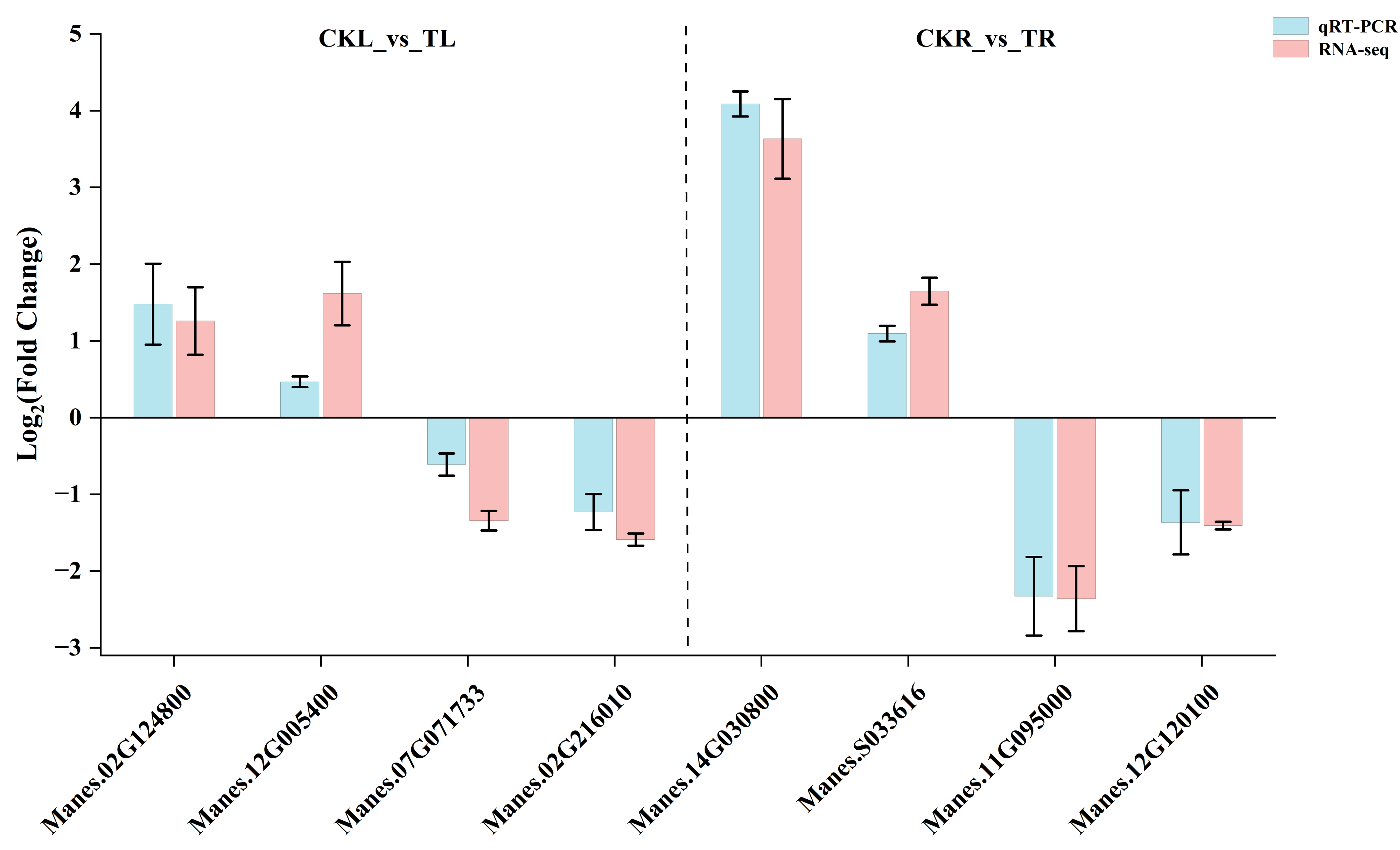


**Supplementary Figure 2.** Validation of transcriptome sequencing results by qRT-PCR. The x-axis represents gene ID, and the y-axis denotes log_2_(Fold Change) values.


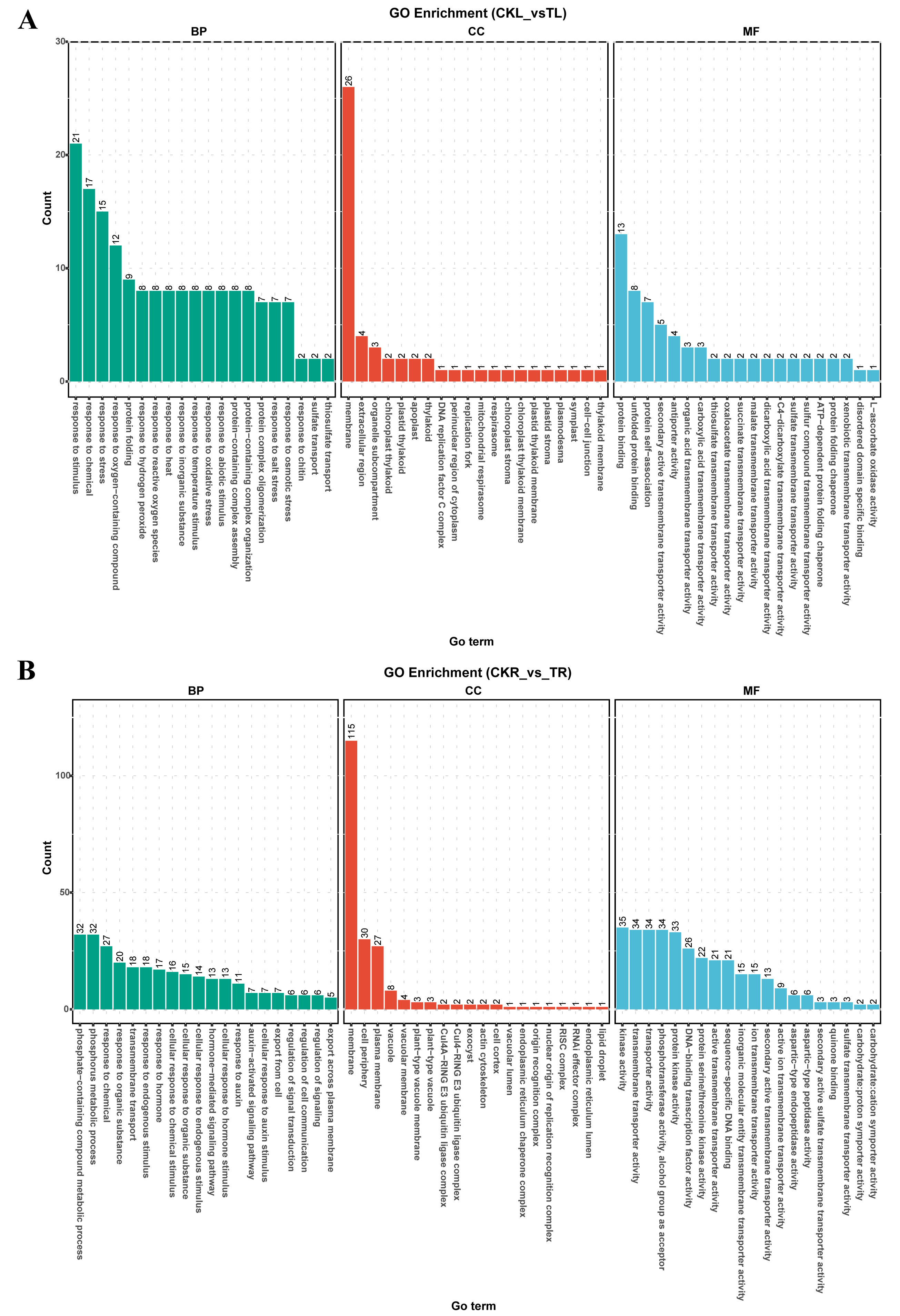


**Supplementary Figure 3.** GO enrichment analysis bar plot of differentially expressed genes. The x-axis represents Gene Ontology terms, the y-axis indicates the number of differentially expressed genes, and colors correspond to functional categories. BP: Biological Process; CC: Cellular Component; MF: Molecular Function. The plot displays only the top 20 differentially expressed genes with the most significant *p*-values in each category.


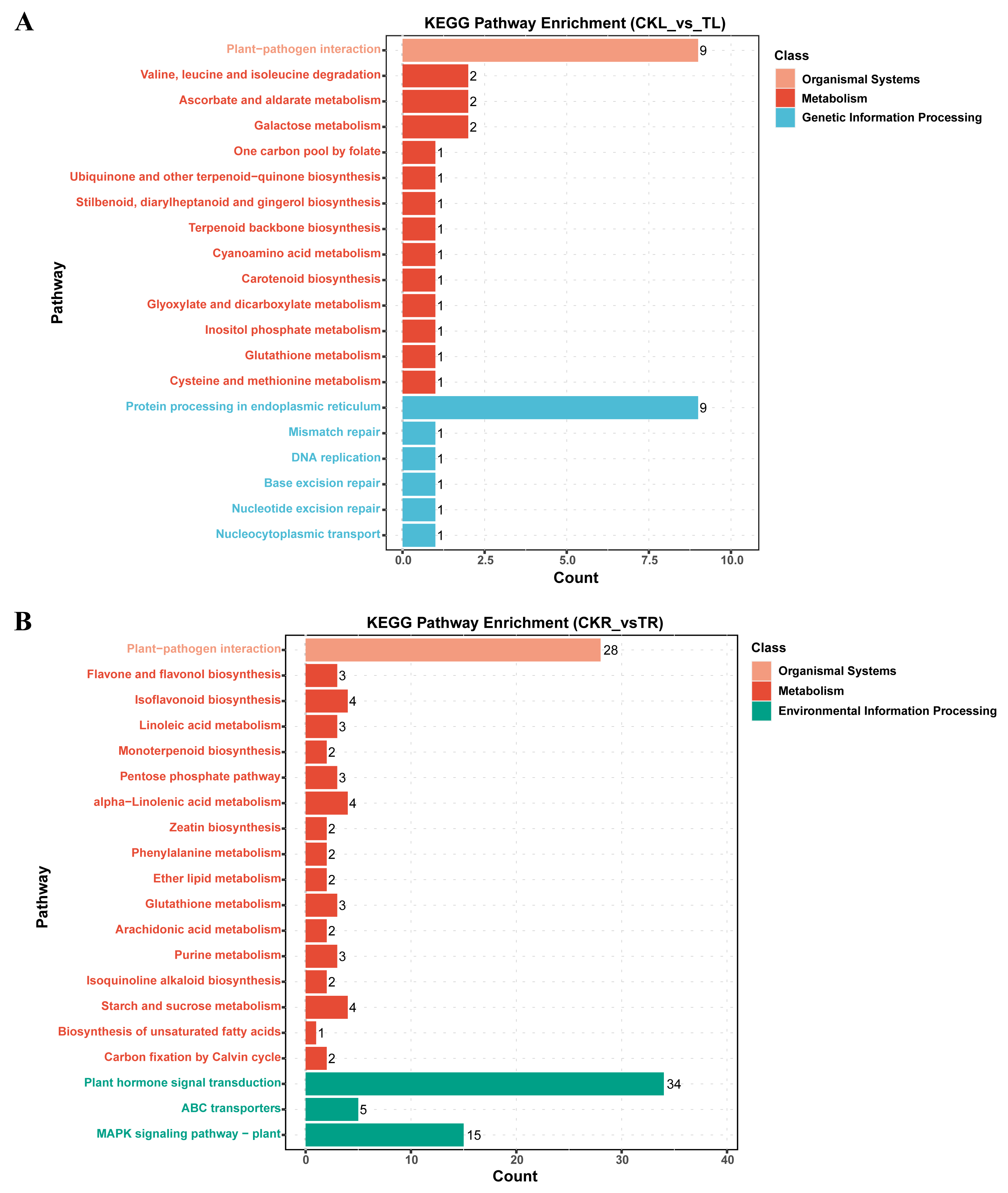


**Supplementary Figure 4.** KEGG enrichment analysis bar plot of differentially expressed genes. The x-axis represents the number of differentially expressed genes, the y-axis denotes KEGG pathway terms, and the colors indicate functional categories.

**
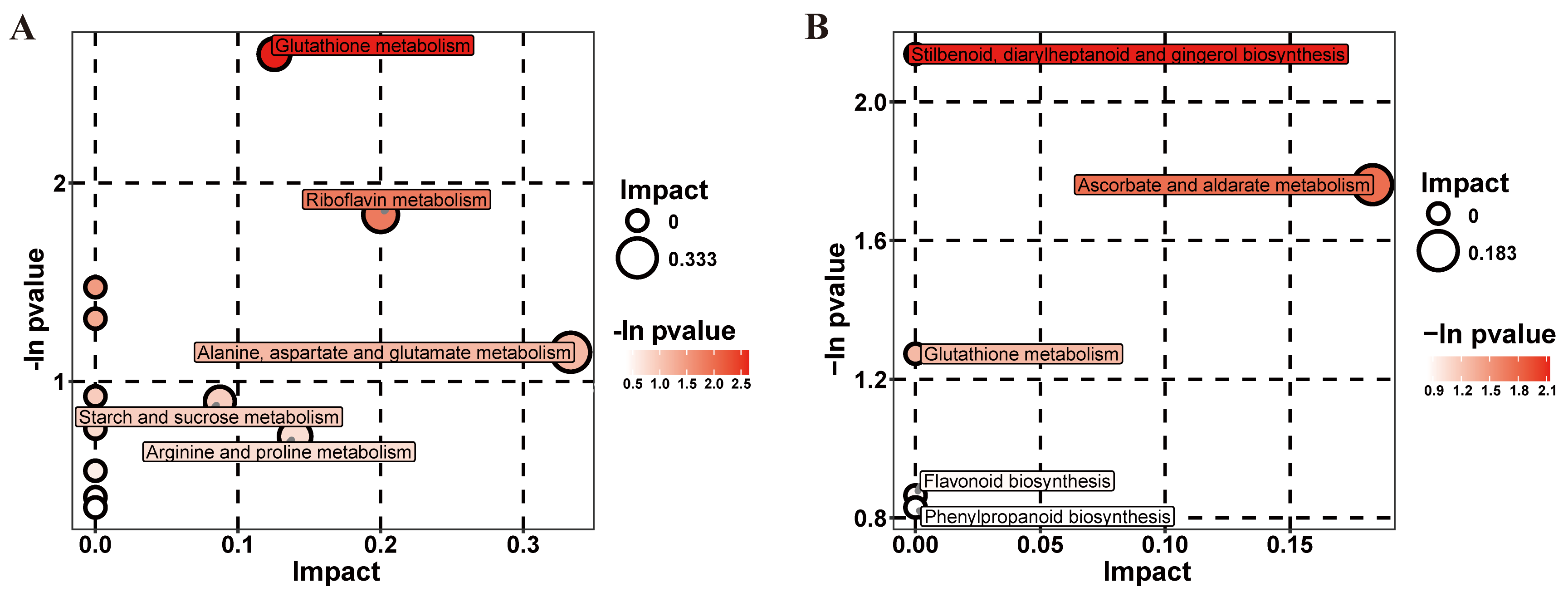
**

**Supplementary Figure 5.** Bubble plots of metabolomic pathway analysis. Each bubble represents a metabolic pathway. The x-axis position and bubble diameter indicate the topological impact factor, while the y-axis position and bubble color correspond to the enrichment-derived −ln(*p*-value). (A) CKL_vs_TL group; (B) CKR_vs_TR group.


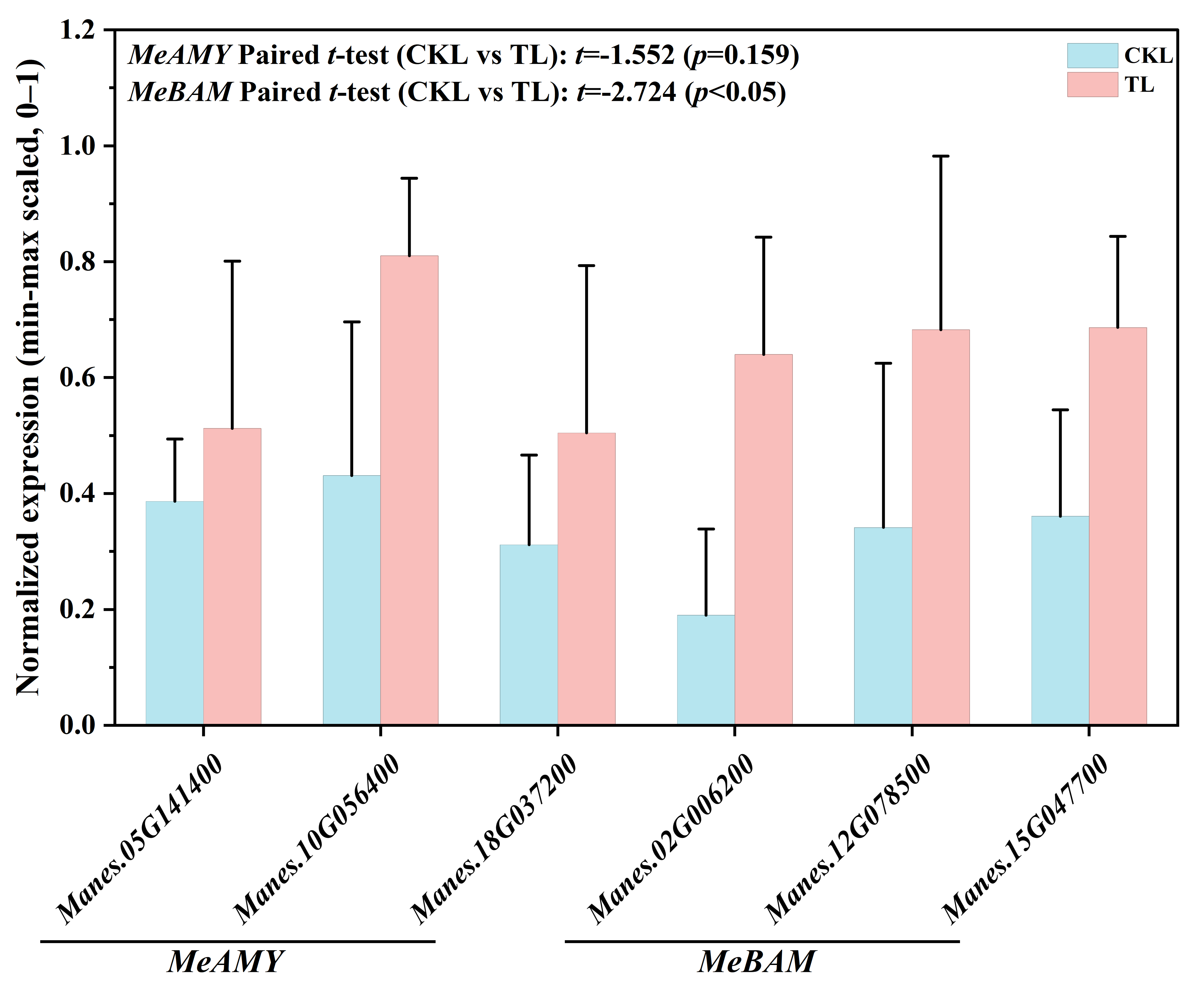


**Supplementary Figure 6.** The relative expression levels of the *MeAMY* and *MeBAM* gene families were analyzed, and paired *t*-tests were conducted to assess differences between treatments. All gene expression data presented in the figure were normalized using min-max scaling, which scales values to a range of 0 to 1.
